# A questionnaire survey on evaluation for penetration and compliance of the Japanese Guideline on Febrile Neutropenia among hematology-oncology physicians and surgeons

**DOI:** 10.1007/s00520-021-06277-8

**Published:** 2021-05-18

**Authors:** Nobu Akiyama, Takuho Okamura, Minoru Yoshida, Shun-ichi Kimura, Shingo Yano, Isao Yoshida, Hiroyuki Kusaba, Kosuke Takahashi, Hiroyuki Fujita, Keitaro Fukushima, Hiromichi Iwasaki, Kazuo Tamura, Toshiaki Saeki, Yasushi Takamatsu, Sadamoto Zenda

**Affiliations:** 1 Department of Internal Medicine, School of Medicine, Teikyo University, Kaga 2-11-1, Itabashi ward, 173-8605 Tokyo, Japan; 2 Department of Breast and Endocrine Surgery, School of Medicine, Tokai University, Isehara, Kanagawa Japan; 3 Fourth Department of Internal Medicine, Teikyo University Hospital Mizonokuchi, Kawasaki, Kanagawa Japan; 4 Division of Hematology, Jichi Medical University Saitama Medical Center, Saitama, Saitama Japan; 5 Division of Clinical Oncology and Hematology, The Jikei University School of Medicine, Minato, Tokyo Japan; 6 Department of Hematologic Oncology, National Hospital Organization Shikoku Cancer Center, Matuyama, Ehime Japan; 7 Department of Comprehensive Clinical Oncology, Faculty of Medical Sciences, Kyushu University, Fukuoka, Fukuoka Japan; 8 Department of Respiratory Medicine, Anjo Kosei Hospital, Anjo, Aichi Japan; 9 Department of Hematology, Saiseikai Yokohama Nanbu Hospital, Yokohama, Kanagawa Japan; 10 Department of Pediatrics, Dokkyo Medical University, Mibu, Tochigi Japan; 11 Department of Infection Control and Prevention, Faculty of Medical Sciences, University of Fukui, Eiheiji, Fukui Japan; 12 Fukuoka University, Fukuoka, Fukuoka Japan; 13 Department of Breast Oncology, Saitama Medical University International Medical Center, Hidaka, Saitama Japan; 14 Department of Hematology, Oncology, Endocrinology and Infectious Disease, Fukuoka University, Fukuoka, Fukuoka Japan; 15 Department of Radiation Oncology, National Cancer Center Hospital East, Kashiwa, Chiba Japan

**Keywords:** Febrile neutropenia, G-CSF, Guidelines, Supportive care, Chemotherapy, Surveillance

## Abstract

**Purpose:**

The Japanese Society of Medical Oncology published a guideline (GL) on febrile neutropenia (FN) in 2017. The study’s purpose is to reveal how widely GL penetrated among physicians and surgeons providing chemotherapy.

**Methods:**

A questionnaire survey was conducted with SurveyMonkey™ for members of the Japanese Association of Supportive Care in Cancer and relevant academic organizations. Each question had four options (always do, do in more than half of patients, do in less than half, do not at all) and a free description form. Responses were analyzed with statistical text-analytics.

**Result:**

A total of 800 responses were retrieved. Major respondents were experts with more than 10-year experience, physicians 54%, and surgeons 46%. Eighty-seven percent of respondents knew and used GL. Forty-eight percent assessed FN with Multinational Association of Supportive Care in Cancer (MASCC) score “always” or “more than half.” Eighty-one percent chose beta-lactam monotherapy as primary treatment in high-risk patients. Seventy-seven percent did oral antibacterial therapy in low-risk patients ambulatorily. Seventy-eight percent administered primary prophylactic G-CSF (ppG-CSF) in FN frequency ≥ 20% regimen. Fifty-nine percent did ppG-CSF for high-risk patients in FN frequency 10–20% regimen. Ninety-seven percent did not use ppG-CSF in FN frequency < 10% regimen. The medians of complete and complete plus partial compliance rates were 46.4% (range 7.0–92.8) and 77.8% (range 35.4–98.7). The complete compliance rates were less than 30% in seven recommendations, including the MASCC score assessment.

**Conclusion:**

GL is estimated to be widely utilized, but some recommendations were not followed, presumably due to a mismatch with actual clinical practices in Japan.

## Introduction

Febrile neutropenia (FN) is a potentially fatal infectious complication in cancer chemotherapy. Various guidelines were published abroad [1–5], some of which were evaluated for compliance with guideline recommendations and clinical outcomes [6–9]. The Japanese guideline (GL) on FN, developed by a multidisciplinary expert panel based on evidence from other guidelines, meta-analyses, and systematic reviews, was published in 2014 and revised in 2017 by the Japanese Society of Medical Oncology and is comparable to internationally accepted ones. This study aims to determine how widely GL is known and followed among physicians and surgeons involved in chemotherapy and identify the causes of gaps between GL recommendations and actual clinical practice. In Japan, surgeons traditionally provide chemotherapy in their specialties.

## Materials and methods

A questionnaire consisting of 21 questions on GL and seven on attributes of respondents was surveyed through SurveyMonkey™ for the members of the Japanese Association of Supportive Care in Cancer, the Japanese Society of Medical Oncology, the Japanese Society of Hematology, and the Japanese Breast Cancer Society (Table 1). Except for Q1, each question had four options: (1) always do, (2) do in more than half of patients, (3) do in less than half of patients, (4) do not at all, and a free description form. The options of Q1 were as follows: (1) have a printed GL and apply it to clinical practice; (2) have a download format of GL and apply it to clinical practice; (3) know GL but do not use it; (4) do not know GL. The responses were retrieved in CSV format and then analyzed with Microsoft Excel. Text mining with KH coder was used to operate co-occurrence analyses and cluster analyses of posted comments [10].

**Table 1:** Questions

Attributes of responders	Gender, a rank of age, year of graduation of medical school, type of institution, subspecialty, board certifications, affiliated academic societies
Questions on GL	
Q1. Do you know the Clinical Guidelines on Febrile Neutropenia revised 2nd version published from the Japanese Society of Medical Oncology in 2017?	
Q2. Do you assess the risk of FN with the MASCC score?	
Q3. Do you take two sets of blood cultures from different body sites at the onset of FN in outpatient care?	
Q4. Do you take two sets of blood cultures from different body sites at the onset of FN in-hospital care?	
Q5. Do you take one set of blood cultures from each of a peripheral vein and a CVC, if indwelled?	
Q6. Do you treat a high-risk FN patient with beta-lactam monotherapy as the first-line therapy?	
Q7. Do you treat a low-risk FN patient with oral antibacterial as the first-line therapy?	
Q8. Do you provide outpatient treatment for a low-risk FN patient?	
Q9. When fever resolves with initial treatment despite persisting neutropenia, do you switch the initial therapy to oral antibacterial or discontinue it?	
Q10. When the patient’s general condition is stable despite persistent FN over 3–4 days after the first-line therapy initiation, do you continue it?	
Q11*. Do you administer therapeutic G-CSF to a patient with FN?	
Q12*. Do you administer intravenous gamma-globulin for a high-risk FN patient?	
Q13. When a patient indwelled with CVC has FN accompanied by either thrombophlebitis, infectious endocarditis, or positive blood cultures of *Staphylococcus aureus*, *Pseudomonas aeruginosa*, *Bacillus* species, and *Candida* species, do you remove the CVC?	
Q14*. Do you practice antibacterial prophylaxis for a patient expected with low-grade neutropenia?	
Q15. Do you use G-CSF as primary prophylaxis in the regimens of FN occurrence of more than 20%?	
Q16. Do you use G-CSF as primary prophylaxis to a patient with a high risk for developing FN in the regimens of FN occurrence between 10 and 20%?	
Q17*. Do you use G-CSF as primary prophylaxis in the regimens of FN occurrence of less than 10%?	
Q18. Do you screen HBV infection, including the measurement of HBs antigen, anti-HBs antibody and anti-HBc antibody before the initiation of cancer chemotherapy?	
Q19. Do you screen tuberculosis, including chest X-ray examination and history taking of the previous infection and recent contact with the patients before starting chemotherapy?	
Q20. Do you practice vaccination of influenza for patients receiving cancer chemotherapy?	
Q21. Do you practice vaccination of *Streptococcus pneumoniae* for patients receiving cancer chemotherapy when they are either between two months and six years of age or older than 65 years old?	

^*^The GL does not recommend those practices

## Results

**Fig. 1:**
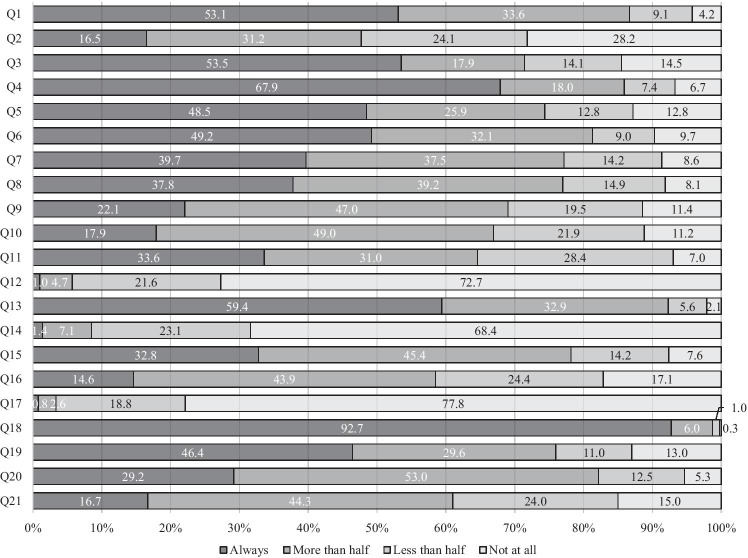
Frequency distribution of choice in each question

Eight hundred and one responses were retrieved. A respondent, a pharmacist, was excluded from all of the analyses. Twelve, including three palliative care specialists and nine radiation oncologists, who are not involve in the treatment of FN, were excluded from the analyses of questions on GL. The characteristics of the respondent and the results of the questionnaire are demonstrated in Table 2 and Fig. 1.

### Q1: penetration and usage of GL

A total of 86.7% knew and applied GL. A total of 9.1% knew but did not apply it. A total of 4.2% did not know it. Twelve comments were posted. Four referred to the main reason for not using or not knowing it as no encounter of FN.

### Q2: risk assessment of FN with the Multinational Association of Supportive Care in Cancer score[11]

A total of 47.7% used the Multinational Association of Supportive Care in Cancer (MASCC) score (MS) to assess the risk of FN “always” or “more than half.” Eighty-nine comments were posted as to why “less than half” or “not at all” and categorized into seven groups: exclusively see high-risk patients, who will be hospitalized when FN occurs, 15 comments; do not calculate the score but refer to the evaluation criteria to roughly assess the risk, 14; judge the risk according to the unique criteria including patient conditions and clinical experience, 14; feel cumbersome, complicated, and unnecessary, 11; see only low risk or do not experience FN, 11; do not know MS, 9; others 15.

### Q3 ~ 5: blood culture

A total of 71.4% and 85.9% took two sets of blood cultures at the onset of FN “always” or “more than half” in outpatient clinics and in-hospital care, respectively. Seventy-six comments on “less than half” and “not at all” in the outpatient setting were categorized into eight: FN patients will be hospitalized and be collected two sets of blood cultures in-hospital care, 13; two sets collection imposes a heavy burden on doctors and medical staffs, 11; when FN occurs, patients will take oral antibacterial at home prescribed in advance, 10; no encounter of FN, 9; do not take blood culture in low-risk patients even if FN occurs, 8; two sets is a heavy burden on patients, especially children, 8; do not implement it as antibacterial treatment is prioritized, 5; others 12. In the case of in-hospital care, 27 posted comments were summarized as follows: a heavy burden on doctors and nurses, 6; no encounter of FN, 5; a heavy burden on pediatric patients, 5; do not take blood culture in low risk, 4; others 7.

A total of 74.4% collected one set of blood cultures from each of the central venous catheter (CVC) and the peripheral vein “always” or “more than half.” Sixty-seven posted comments on “less than half” and “not at all” were categorized into eight: no encounter of the applicable cases, 22; blood drawing from CVC is considered meaningless, 10; collect blood cultures only from the peripheral vein (unspecified reasons), 9; CVC will be removed in suspicion of catheter-related infection, 6; as a central venous port is installed if necessary, it is hard to draw blood from the port, 5; collect blood cultures only from CVC (unspecified reasons), 4; two sets collection is a heavy burden on adult patients as well as pediatric, 3; others 8.

### Q6 ~ 8: the primary therapy

A total of 81.3% administered intravenous beta-lactam monotherapy for the high-risk FN patients “always” or “more than half” as the first-line therapy. Twenty-six comments on “less than half” or “not at all” were summarized as follows: no encounter of applicable cases, 8; use fluoroquinolone, 5; practice combination therapy (unspecified), 2; others 11 including four “use carbapenem” due to misunderstanding.

A total of 77.2% administered oral antibacterial for the low-risk FN patients, and 77.0% saw those patients ambulatorily “always” or “more than half”. Forty-one and 43 comments on Q7 and Q8 for “less than half” and “not at all” were divided into three groups with the same titles each: an FN patient needs to admit and to take intravenous antibacterial in-hospital care, 30 and 37; intravenous antibacterial is administered in-hospital care because it is difficult for pediatric patients to take oral antibacterial, 4 and 2; others 7 and 4, respectively.

### Q9 ~ 13 the management of FN

A total of 69.1% “always” or “more than half” switched the initial intravenous antibacterial to oral agents or discontinued it when fever resolved with initial treatment despite persisting neutropenia. Forty comments on “less than half” and “not at all” were categorized into five: continue intravenous antibiotic until neutrophil recovery (unspecified reasons), 21; do not encounter applicable patients, 8; neutrophils usually recovered concurrently with defervescence, 4; decide to either continue primary therapy or cease it or shift to oral antibacterial according to the patient’s condition, 4; oral antibacterial is improper in pediatric FN patients for insufficient evidence or poor adherence, 3.

If the patient’s general condition is stable despite the persistence of FN over 3–4 days after the initiation of the first-line therapy, 66.9% continued it. Ninety-six comments on “less than half” and “not at all” were categorized into four: shift to another antibacterial or add an anti-methicillin-resistant *Staphylococcus aureus* agent or antifungal to the first-line therapy in consideration of treatment failure, 73; decide to either continue the primary treatment or change it based on scrutiny, 9; no encounter of applicable patients, 7; others 7.

Only 35.4% did not use G-CSF for therapeutic purposes in an FN patient, or “less than half” use G-CSF, as GL does not recommend using it. Thirty-eight comments on “always” and “more than half” were categorized into seven: expect recovering from neutropenia as soon as possible to shorten hospital stay and reduce patient burden, 10; use G-CSF in the high-risk FN patients, 7; use G-CSF for solid tumors and lymphoid tumors but not for acute myeloid leukemia, 5; prevent deteriorating of FN, 4; use G-CSF in the case of profound neutropenia, 4; others 8.

A total of 94.3% did not use or “less than half” use intravenous gamma-globulin in the high-risk FN patients, as GL does not recommend it. Twelve comments on using it “always,” “more than half,” and “less than half” were summarized as follows: use it in the FN patients with severe conditions, 5; administer it to the patients with hypogammaglobulinemia, 4; others 3.

A total of 92.3% “always” or “more than half” removed CVC when patients had FN accompanied by either thrombophlebitis, infectious endocarditis, or positive blood cultures of *Staphylococcus aureus*, *Pseudomonas aeruginosa*, *Bacillus* species, and *Candida* species. Twenty-one comments on “less than half” and “not at all” were summarized as follows: it is hard to remove the central venous port, 4; re-insertion of CVC is challenging, 3; CVC is retained in the patients with the difficulty of intravenous catheterization, 3; no encounter of applicable patients, 6; others 5.

### Q14 ~ 17 the prevention for FN

A total of 91.5% did not practice, or “less than half” practiced oral antibacterial prophylaxis in patients with low-grade neutropenia because GL does not recommend it. Seven comments on “always” and “more than half” varied but included one “Sulfamethoxazole/trimethoprim tablet is used to prevent *Pneumocystis* pneumonia.”

A total of 78.2% used G-CSF as primary prophylaxis (ppG-CSF) “always” or “more than half” in the regimens of FN occurrence of more than 20%. Forty-four comments on “less than half” and “not at all” were categorized into seven: apply ppG-CSF in consideration with the chemotherapeutic regimen and patient’s condition, 11; examine whether prophylactic G-CSF is needed based on neutrophil counts during the first course of chemotherapy, 10; do not need to use ppG-CSF because of no encounter of applicable patients, 9; use G-CSF prophylactically from the following course if FN develops, 4; it is not profitable to use pegfilgrastim in-hospital care due to the diagnosis procedure combination (DPC)-based payment system, 3; reduce the doses of antineoplastic agents instead of administering prophylactic G-CSF, 2; others 5.

A total of 58.5% used ppG-CSF “always” or “more than half” to the high-risk patients for FN in the regimens of FN occurrence of 10–20%. Fifty-four comments on “less than half” and “not at all” were categorized into eight: examine whether prophylactic G-CSF is needed based on neutrophil counts during the first course of chemotherapy, 14; do not use ppG-CSF due to unprofitability in DPC, 9; use G-CSF prophylactically from the following course if FN develops, 8; do not need to use ppG-CSF because of no encounter of applicable patients, 6; apply ppG-CSF in consideration with patient’s condition, 6; reduce the doses of antineoplastic agents or change regimen instead of administering prophylactic G-CSF, 2; administer prophylactic oral antibacterial, 2; others 7.

A total of 96.6% did not use or “less than half” use ppG-CSF in the regimens of FN occurrence of less than 10% because of no recommendation in GL. Twelve comments on “always” and “more than half” were summarized as follows: consider to use it up to the patient’s conditions, 8; use it in the patients with a history of severe FN, 2; others 2.

### Q18 and 19 screening for hepatitis B virus and tuberculosis

A total of 98.7% “always” or “more than half” screened HBV infection, including the examination of HBs antigen, anti-HBs antibody, and anti-HBc antibody before the initiation of chemotherapy.

A total of 76.0% “always” or “more than half” screened tuberculosis, including chest X-ray examination and taking a history of the previous infection and recent contact with a patient with tuberculosis before the initiation of chemotherapy. Twenty-eight comments on “less than half” and “not at all” were summarized as follows: examine chest X-ray or CT but do not take the history of recent contact with a tuberculosis patient, 19; others 9.

### Q20 and 21 vaccination

A total of 82.2% practiced “always” or “more than half” influenza vaccination for patients receiving chemotherapy. Forty-eight comments on “less than half” and “not at all” were categorized into five: the efficacy of vaccination does not look promising during chemotherapy, in particular including immunosuppressants like rituximab and corticosteroids, 16; it is up to the patient to decide whether or not to get the vaccination, 13; vaccination is recommended but is not provided in the hospital, 6; chemotherapy is prioritized to vaccination, 3; others 10.

A total of 61.0% practice “always” or “more than half” vaccination of *Streptococcus pneumoniae* for patients receiving chemotherapy when they are either between 2 months and 6 years of age or older than 65 years old. GL recommends 13 valent and 23 valent vaccines for the former and the latter, respectively. Seventy-four comments on “less than half” and “not at all” were categorized into eight: it is up to a patient to decide whether or not to get the vaccination, 20; let the family doctor determine whether or not to provide the vaccination, 13; vaccination is given after recovering from the immunosuppression caused by chemotherapy, 7; the efficacy of vaccination does not look promising during chemotherapy, 7; vaccinate if a patient gets a public subsidy, 6; have never been aware of it, 6; chemotherapy is prioritized to the vaccination, 3; others 12.

**Table 2:** Characteristics of respondents

Characteristics	The number of respondents (*N* = 800)	
Gender	Men to women ratio: 622 to 178	
A rank of age	The mode of age rank: 40–44 years old	151 (18.9%)
	From 35 to 64 of age	724 (90.5%)
Type of institution	University hospital	290 (36.2%)
	Cancer center hospital	307 (38.4%)
	General hospital	176 (22.0%)
	Outpatient clinic	11 (1.4%)
	Others	16 (2.0%)
Specialty	Physicians	434 (54.25%)
Subspecialty	Hematology	118 (14.7%)
Board certification	Board-certified hematologists	113
	Non-hematology*	316 (39.5%)
	Board-certified medical oncologists	195
	Medical oncology	197
	Pulmonary medicine	103
	Gastroenterology	70
	General medicine	4
	Pediatrics	10
	Breast medicine	17
	Radiation oncology	9
	Palliative care	12
	Surgeons	366 (45.75%)
	Breast surgery	287 (35.9%)
	Board-certified breast surgeons	241
	Non-breast surgery*	79 (9.9%)
	Board-certified medical oncologists	19
	Board-certified general clinical oncologists^#^	50
	Gastroenteric surgery	28
	Thoracic surgery	6
	General surgery	17
	Obstetrics	20
	Urology	5
	Otorhinolaryngology	5
	Orthopedics	6
	Others	3

^*^It redundantly comprised of the following subspecialties

^#^There are two different board certifications for chemotherapy: medical oncologists accredited by the Japanese Society of Medical Oncology and general clinical oncologists accredited by the Japanese Board of Cancer Therapy

### Compliance with GL

Each question is about what GL recommends, except for Q11, 12, 14, and 17. The responses to those questions represent the degree of compliance with GL. “Always do” and “do in more than half of patients” suggest complete and partial compliance, respectively. In contrast, so do “do not at all” and “do in less than half of the patients” in Q11, 12, 14, and 17. The medians of the complete and complete plus partial compliance rates were 46.4% (range 7.0–92.8) and 77.8% (range 35.4–98.7), respectively. The complete compliance rates were less than 30% in seven recommendations (Q2, 9–11, 16, 20, and 21).

## Discussion

### The validity of the questionnaire

The cumulative total numbers of the doctor members of each academic society were 21,252 consisting of 510 in the Japanese Association of Supportive Care in Cancer, 6900 in the Japanese Society of Medical Oncology, 6180 in the Japanese Society of Hematology, and 7662 in the Japanese Breast Cancer Society. Since some of the respondents belong to two or more societies, the estimated number is considered more than actual persons. Even if the number of target population is 100,000, sample size is calculated as 383 under the condition of a 5% margin of error and 95% confidence level [12]. Therefore, the numbers of responses are sufficient to assume that the results represent the population’s opinions with a 5% margin of error. Besides, respondents are regarded as experts for chemotherapy because of over 90% with more than 10-year experience, and three-fourths belonging to university hospitals and cancer center hospitals.

### Penetration, state of usage, and disclosed problems of GL

It has become evident that GL is well known and used in daily clinical practices. Concerning that 95.8% knew GL, however, it cannot be ruled out that the respondents may have been biased toward who were familiar with GL, as they voluntarily participated in this survey. The state of complying with GL is considered acceptable, as the complete plus partial compliance rate was just under 80% in the majority of the recommendations. However, the complete compliance rates were lower than expected, and those in some of the recommendations were less than 30%. Through this survey, major four problems responsible for the quality of FN management have been identified: risk assessment of FN (Q2), primary therapy for FN in low-risk FN patients (Q7 & 8), management of FN (Q9 & 10), usage of G-CSF (Q11, Q15, and 16).

### Risk assessment of FN with MS

GL weakly recommends estimating FN risk with MS at the onset. It was inherently designed to identify low-risk patients [11]. Risk assessment is required when an FN patient will be managed ambulatorily. The complete plus partial compliance of the risk assessment was low as 47.7% even though 77.0% responded “always” and “more than half” to provide outpatient treatment for a low-risk FN patient. It is acceptable not to assess the risk for the respondents who exclusively see high-risk patients requiring hospitalization. However, respondents who evaluated the risk based on their clinical experience or who did not apply the score because they felt cumbersome or unnecessary should not be overlooked.

On the other hand, MS has been pointed out to have a couple of problems as about 10% of patients who are identified as low risk will develop severely ill. The American Society of Clinical Oncology and Infectious Disease Society of America guidelines for the outpatient management of FN advocate combining it with the Clinical Index of Stable Febrile Neutropenia (CISNE) score to increase the determination accuracy [2]. It is crucial to make evidence-based decisions on whether or not a patient needs in-hospital care and intravenous antimicrobial as primary treatment. Concurrently, the risk assessment criteria may need to be amended considering the healthcare environment in Japan.

### Primary therapy

GL strongly recommends intravenous beta-lactam monotherapy for high-risk FN patients. The overall compliance rate, meaning as complete plus partial compliance rate, of 81.3% was deemed adequate. GL weakly recommends both oral antibacterial therapy and management in the outpatient setting for low-risk FN patients. The overall compliance rates for these two recommendations were just below 80% and considered reasonable. However, the primary therapy’s complete compliance rates in high- and low-risk patients were considerably low, 49.2% and 39.7%, respectively. A survey of 25,231 admitted FN cases in the USA revealed that 79% received guideline-based antibiotics [6]. Two surveillances of GL adherence in the emergency departments demonstrated that the GL concordant management rates were substantially high of 96.8% and 98% in high-risk FN patients; in contrast, those were extremely low of 0.4% and 2% in low-risk patients [7, 8]. And the cause of non-compliance in the low risk was overtreatment [7, 8]. Since the present study cannot identify whether over- or under-treatment, GL’s adherence based on cases should be surveyed to clarify if suitable treatment is selected according to patients’ risk.

### The management of FN

The GL weakly recommends switching from the initial intravenous antimicrobial to an oral agent or discontinuing it if the fever subsides after the initial treatment despite persistent neutropenia. Besides, if the patient’s general condition is stable regardless of persistent FN for more than 3–4 days after initiation of therapy, it is weakly recommended to continue the primary treatment. Although the overall compliance rates were good, the complete compliance rates, 22.1% and 17.9%, were substantially low. Many respondents probably thought that intravenous antimicrobials should be continued until neutrophil recuperate because discontinuation may cause a resurgence of infection, and that persistent fever, even in stable condition, was deemed to be treatment failure. To comply with these recommendations presumably depends on multifactor, including patient condition, local healthcare-providing system, the evidence level, and healthcare economy. According to the questionnaire survey on antimicrobial practice for FN across European and Asian blood and marrow transplantation centers, about half of the centers responded to de-escalate or discontinue the first-line therapy when the fever of unknown origin with an uncomplicated presentation resolved by the treatment [9]. The disincentives should be investigated in more detail. Stewardship of appropriate antimicrobial use for FN needs to be established in the era of multidrug-resistant bacteria.

### G-CSF

GL does not recommend therapeutic G-CSF administration, which means starting G-CSF after FN onset, and advises to consider its application in case of deterioration. The rates of therapeutic G-CSF “always” and “more than half” were 33.6% and 31.0%, respectively. A case-based survey disclosed that 63% of hospitalized FN patients were administered therapeutic G-CSF in the USA [6]. Although we cannot estimate the actual percentage of patients receiving G-CSF, the results are considered comparable to the previous study. The primary reason for administering it was expecting faster recovery from neutropenia. It seems acceptable, but this suggests that patients who must have needed prophylactic G-CSF did not receive it. Prevention measures should be primarily taken for FN since it is a potentially fatal complication and may result in dose reduction or treatment delay in chemotherapy and eventually jeopardize overall survival [13, 14].

Although GL strongly recommends using ppG-CSF in the regimens with FN occurrence of more than 20%, the complete compliance rate was low as 32.8%. Besides, regarding the use of ppG-CSF in patients at high risk for developing FN in regimens with FN incidence of 10–20%, the overall and complete compliance rates were also low as 58.5 and 14.6%, respectively. These results indicate that the ppG-CSF was discouraged by several disincentives, including low profitability in the DPC. A questionnaire survey of oncologist’s perceptions and opinions regarding the use of G-CSF in the USA demonstrated that 65% agreed using ppG-CSF in all of the patients with high risk for developing FN [15]. It also revealed the common barriers to G-CSF use, including insurance-related matters. Case-based surveys on the adherence to G-CSF guidelines revealed that the adherence rates were 43% [16], 73.1% [17], 76.6% [18], and 79.0% [19] for ppG-CSF in the patients receiving FN high-risk regimens. Belgian surveillance of moderate- and high-risk FN in breast cancer and non-Hodgkin lymphoma demonstrated that ppG-CSF was administered in less than 1% of breast cancer and 26% of non-Hodgkin lymphoma, the incidence of FN was higher in the patients without ppG-CSF, and chemotherapy delivery (timing and/or dose) was impaired in about 40% of patients developing FN [20]. The complete compliance rate for ppG-CSF in the present study was considered lower than the previous reports. Further investigation is needed to remove disincentives and to promote the appropriate use of G-CSF.

The present study clarified the discrepancy between GL recommendations and daily practice perception in the doctors engaging in chemotherapy. Due to the study’s limitations, these results could deviate from the actual practices when analyzing the patients’ files. To promote evidence-based FN management, further research will be needed, including prospective or retrospective cohort studies to disclose GL compliance state and questionnaire to identify inhibitory factors causing the gaps.

## Data Availability

Analyses and text mining were carried out using Microsoft Excel 2019 and KH-Coder, which is a freeware developed by K Higuchi, Ritumeikan University [10].
